# Teens Implementing a Childhood Obesity Prevention Program in the Community: Feasibility and Perceptions of a Partnership with HSTA and iCook 4-H

**DOI:** 10.3390/ijerph15050934

**Published:** 2018-05-07

**Authors:** Rebecca L. Hagedorn, Jade A. White, Lisa Franzen-Castle, Sarah E. Colby, Kendra K. Kattelmann, Adrienne A. White, Melissa D. Olfert

**Affiliations:** 1 Natural Resources & Design, Division of Animal and Nutritional Sciences, Davis College of Agriculture, West Virginia University, G016 Agricultural Science Building, Morgantown, WV 26506, USA; rlhagedorn@mix.wvu.edu (R.L.H.); jade_white@my.uri.edu (J.A.W.); 2 Nutrition and Health Sciences Department, University of Nebraska-Lincoln, 110 Ruth Leverton Hall, Lincoln, NE 68583, USA; lfranzen2@unl.edu; 3 Department of Nutrition, University of Tennessee, 1215 W. Cumberland Avenue, 229 Jessie Harris Building, Knoxville, TN 37996, USA; scolby1@utk.edu; 4 Department of Health and Nutritional Sciences, South Dakota State University, Box 2275A, SWG 425, Brookings, SD 57007, USA; kendra.Kattelmann@sdstate.edu; 5 School of Food and Agriculture, University of Maine, 5735 Hitchner Hall, Orono, ME 04469, USA; awhite@maine.edu

**Keywords:** teens as teachers, high school, childhood obesity

## Abstract

High school student researchers and teen leaders from the Health Science Technology Academy (HSTA), under the supervision of HSTA teachers, led a childhood obesity prevention (COP) program (iCook 4-H). The objective was to evaluate the feasibility and perceptions of having teen leaders implement a COP program for dyads of youth (9–10 years old) and their primary adult food preparer. Behavior change and perceptions were assessed through surveys and open-ended interviews. Across eight HSTA organizations, 43 teen leaders participated in teaching the iCook 4-H program to 24 dyads. Increased frequency of culinary skills, physical activity and mealtime behavior were reported by youth. Almost all adults (93%) reported that their youth had learned kitchen skills and that the program provided youth-adult quality time and developed culinary skills. Youth echoed adult perceptions with additional themes of food safety and physical activity. HSTA teen leaders perceived the program to be successful and reported the training they received to implement the program was adequate 98% of the time. HSTA teachers found the program to be beneficial for HSTA students in improving leadership, confidence and responsibility. iCook 4-H was feasible to be disseminated through teen leaders in the HSTA program. This teen-led approach could serve as a model for youth health-related programming.

## 1. Introduction

The obesity epidemic in the United States has been a paramount focus this past decade, especially regarding the younger generation. Regardless of numerous public health initiatives, obesity rates in youth continue to rise [1]. Obesity is associated with negative health effects for children and adults but, to a child who is still physically and psychologically growing, the side effects of obesity can be more difficult to quantify [2]. Childhood obesity consequences have been studied heavily and show long-term effects on children including clinical issues such as hypertension, type 2 diabetes, inflammation, sleep apnea and self-esteem issues [3,4,5,6,7]. Obese children are also more likely to become obese adults with higher morbidity and mortality rates [8,9,10,11]. Consequently, without intervening, weight gain and obesity will likely continue to result in long-term negative health consequences.

Although childhood obesity is present nationally, researchers have reported that certain regions are more affected by the obesity epidemic than others [4,12]. One such region is Appalachia, which includes thirteen states from New York to Georgia, with West Virginia being in the center and the only state completely engulfed by the Appalachian Mountains. Children of Appalachia show higher prevalence for obesity and other chronic diseases than children from non-Appalachian regions [4,13]. For example, girls in West Virginia have a twofold risk of obesity compared to children in Oregon, a non-Appalachian state [14]. With the childhood obesity rates remaining elevated in underserved areas, such as the Appalachian region, the health of this region’s future generation, is of concern.

Many programs have been developed to address the childhood obesity crisis. These programs are usually developed as adult-led; however, peer-led interventions have also been found to be effective [15]. Peer-led education programs are defined as young people educating other same age people through sharing knowledge and experiences, whereas adult-led programs focus on experts educating younger individuals with adults being the decision makers [16,17]. Teen peer-led programs have gained stronger support in recent years with the development of programs such as the Youth Empowerment Strategies (YES) and the Teens Eating for Energy and Nutrition at School (TEENS) study, which showed success on a teen-teen peer basis [18,19]. In West Virginia, the teen led model has gained success through peer obesity programs such as KEYS 4 Healthy Kids (KEYS = Knowledge, Eating healthy, Youth being active, Safety and empowerment) [20].

While utilizing teens to engage peers has been successful [11], these efforts could be expanded further through teens teaching younger youth to address and/or prevent childhood obesity. The use of cross-age teaching is promoted in adolescents and can expand upon the social learning theory for behavior change in youth [21,22,23,24]. This has the potential to impact the perceptions and behaviors of each of the layered ages involved; youth, adolescent and adult. This ideal is often displayed through the 4-H model “Teens as Teachers”. 4-H clubs across the country have developed programs that engage teens to teach youth as a way to assist in adolescent development [25,26,27,28]. 4-H youth could be viewed as exceptional adolescents as they are more highly trained on specifically developing life skills that many non-4-H teens may not receive specific training on, such as on decision making, psychomotor skills, problem solving, managing resources, communication, and leadership [29,30,31,32]. Therefore, teens involved in 4-H programs may be especially suited to deliver curriculum to younger youth using a “teens as teachers” model. However, while this “Teens as Teachers” model has been successful in the 4-H context with teen volunteers, and in nutrition education programming [33], it is not widely used in other adolescent areas such as teen research programs.

Therefore, the objective of this study was to examine the effectiveness of a “Teens as Teachers”, cross-age approach using teens from the Health Science and Technology Academy (HSTA) in delivering a childhood obesity prevention research program, iCook 4-H [34,35,36]. HSTA is a West Virginia exclusive, high school program that was developed in 1994 for underrepresented students. Eligibility is based on family income, ethnicity, rural status, and first-generation college student status. The goal of HSTA is to provide training and exposure to science, technology, engineering and mathematics (STEM) based programs with the aim of promoting future careers in STEM fields. Through completion of the HSTA program (freshman-senior year of high school), students are provided a tuition waiver to any WV higher education institute. The HSTA program was developed with the expectation that students, after obtaining a higher education degree, would remain in their local communities to promote health and economic development for the state [37,38,39].

Therefore, as a program that promotes health awareness and community engagement, it is an ideal avenue for the dissemination of a childhood obesity prevention (COP) program, such as iCook 4-H. The researchers investigated the feasibility of using HSTA teens, under the supervision of HSTA teachers, to implement a 4-H modeled curriculum for COP programming and explored the perceptions of program (youth-adult dyad) participants, teen HSTA leaders, and adult HSTA teachers.

## 2. Materials and Methods

### 2.1. Study Design

**Figure 1:**
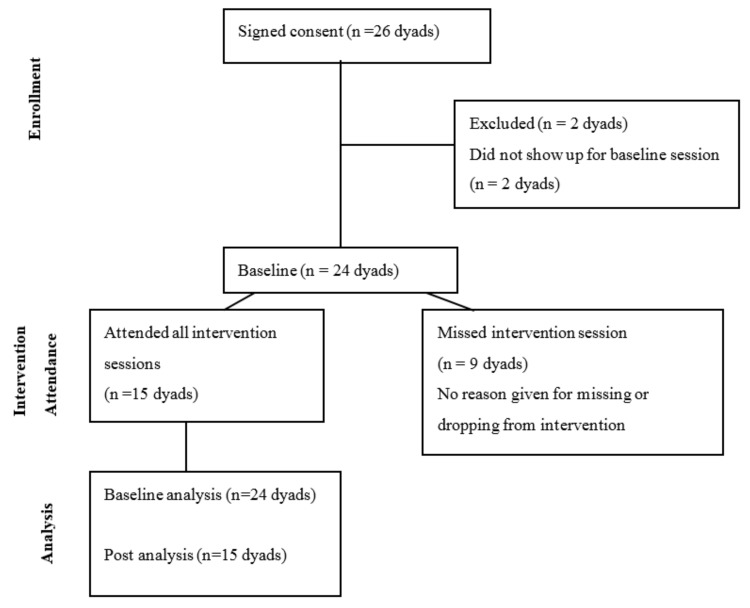
Dyad participation flowchart.

This study was a mixed-methods, exploratory evaluation to test the feasibility of using HSTA teens to disseminate the iCook 4-H program, a childhood obesity prevention program for youth (9–10 years old) and their primary adult food preparer. The program was focused on the promotion of cooking, eating and playing together for healthful lifestyles. Semi-structured interviews and surveys were collected from participants at baseline, during and post intervention. Additionally, HSTA teen leaders and HSTA teachers completed interviews after the intervention. The iCook 4-H program was delivered in eight West Virginia high schools. Between the eight schools, a total of 43 teens were involved in delivering the iCook 4-H curriculum to a total of 24 youth-adult dyads. Initially 26 dyads signed consent forms with 24 participating in baseline assessments. Of the baseline dyads, 62.5% (*n* = 15) remained active throughout all eight iCook sessions and completed post assessments. A participant flowchart is represented in Figure 1.

This study was conducted in accordance with the Declaration of Helsinki and the protocol was approved by the Institutional Review Board at West Virginia University (#1305044336). All researchers and HSTA teen leaders completed the appropriate Collaborative Institutional Training Initiative (CITI) trainings. HSTA teen leaders and teachers and iCook 4-H dyad participants all completed the appropriate informed consent and media releases before participating in the study. Adult supervision from HSTA teachers was present at all times to ensure the safety of dyad participants.

### 2.2. Training

Training for the iCook 4-H program began with HSTA teachers. All HSTA teachers from participating schools were introduced to the curriculum, training platforms and procedures for training HSTA students during an hour-long, in-person training by researchers. The in-person training was followed up by a conference call with iCook 4-H program researchers to provide more detailed step-by-step program procedures on training the HSTA teen leaders. Teachers were then sent training materials through the mail to use to train the HSTA teens to deliver the program. HSTA teachers divided HSTA teens into groups of three teens per group to review their roles as session leaders and become familiar with the training materials prior to the start of the program. The training materials included leader guides for all eight iCook 4-H sessions, quizzes to ensure the teens learned the curriculum before implementation, instruction sheets, online videos and resource posters as well as leader t-shirts and a video camera to record the sessions. All the training materials were also available in an online training platform, eXtension Campus, to provide ease of access for students and teachers. Training continued for each session biweekly that included specific descriptions of the responsibilities of each HSTA teen leader for that specific session.

### 2.3. Intervention Content

The intervention curriculum, iCook 4-H, consisted of three major concepts; cooking, eating and playing together. A description of the iCook 4-H sessions is provided in Table 1 and represents iCook 4-H curriculum used in the pilot dissemination phase [40]. Each session, one through eight, had a culinary skill, physical activity and family meal time component. The HSTA teens were instructed to conduct sessions biweekly, using the off week to learn and prepare. The iCook 4-H sessions were conducted in HSTA clubs between September 2015 and March 2016, with schedules varying by club. During the sessions, HSTA teens had different leadership responsibilities for different aspects of the curriculum, so that each teen had opportunities to gain leadership skills. The HSTA teen in charge of the cooking portion was in charge of leading youth-adult dyad teams through a culinary skills lesson that provided participants the knowledge and tools needed to cook as a family. The HSTA teen in charge of the eating component of the session led a discussion while eating the food prepared during the cooking session, with a goal of promoting positive family meal time and communication. For the playing together element, a HSTA teen led dyads through fun physical activities that could be played together at home.

### 2.4. Measures

#### 2.4.1. iCook Youth Participant Behavior Change

Data was collected at baseline and post intervention for youth participant behavior change. Youth completed a 46-item program outcome tool to assess changes in cooking, eating and playing together behaviors. Survey questions were developed from previous literature and a review of the curriculum content [41]. Reponses were based on 5-point Likert scales, with responses ranging from “never” to “all the time”. Responses “often” and “all the time” (i.e., often/all the time) and “never” and “rarely” (i.e., never/rarely) were combined for analysis of change from baseline to post-assessment.

#### 2.4.2. Dyad Program Perceptions

Dyad program perceptions were collected during and at the completion of the intervention. Youth and adult participants completed a 15-item evaluation that had a combination of 10 Likert scale questions, three open-ended questions, with remaining questions pertaining to the HSTA club and session description (i.e., location, session number completed). Using Likert scale questions, participants responded about applying the lessons learned in the iCook 4-H sessions at home with responses ranging from “never” to “all the time”, “strongly disagree” to “strongly agree” or “very unlikely” to “very likely”. In open-ended questions, participants reported on the most important part of the iCook 4-H program.

#### 2.4.3. HSTA Teen Leader Feedback

HSTA teen leader feedback was collected during and at the completion of the intervention with HSTA teen leaders completing a 12-item leader process evaluation at the completion of each session. Leaders’ reported on their perception of sessions and effectiveness of curriculum materials. Post program, HSTA teen leaders also completed open-ended questions regarding their perception of what were the most beneficial aspects of the program for the dyads.

#### 2.4.4. HSTA Adult Teacher Perceptions

Program feedback was gained from HSTA supervising teachers through open-ended questions by email and semi-structured video interviews at the end of the intervention. The teachers were asked to provide what they thought was beneficial to the dyads and any thoughts on what worked or needed improved in the curriculum for teen leaders.

### 2.5. Analysis

All analysis took place after the completion of the intervention. Written response data were grouped by participant type (youth participant, adult participant, HSTA teen leader, HSTA teacher) for analysis. Video interview data from HSTA teachers were transcribed verbatim by a researcher. Thematic analysis was used to analyze the data with a code book developed to represent codes, definitions and examples following the thematic analysis steps outlined by Braun and Clarke (2006) [42,43]. An initial researcher read all responses noting initial ideas before generating codes for each response. These codes were combined into themes with similar content. These themes were used to represent main messages expressed by iCook 4-H youth and adult participants, HSTA teen leaders and HSTA supervising teachers. Independent of the first researcher, a second researcher evaluated the responses and confirmed the themes that were extracted from the data. If any disagreement arose, both researchers reviewed the data and discussed for further validation, with discrepancies resolved. Descriptive and frequency analysis were also used to summarize the sample population. Paired *t*-test was conducted to detect statistical differences from baseline to post intervention for youth behavior change. Level of statistical significance was set at *p* < 0.05.

## 3. Results

### 3.1. Youth Participant Behavior Change

As presented in Table 2, slight changes were seen from baseline to post-intervention on different behaviors related to cooking, eating and playing together. Culinary skill behavior increased significantly for use of a blender (*p* = 0.0407) at post intervention. Other culinary skills increased but not to statistical significant improvements including ability to use a knife (*p* = 0.0849), ability to use an oven (*p* = 0.3334), plan a meal with all food groups (*p* = 0.4546) and cook to the right temperature (*p* = 0.3572). Being physically active for at least 60 min per day slightly increased from baseline to post (*p* = 0.8845), although families played together less (*p* = 0.5460). Family mealtime behavior improved with improvements in stressful mealtimes (*p* = 0.3673) and eating without distractions (*p* = 0.0777) after the intervention, although not statistically significant.

### 3.2. Dyad Program Perceptions

About 70% of youth reported they completed a fun iCook 4-H activity at home, stated they had a family meal together during the week between sessions and reported they were physically active at home for at least 60 min each day. When asked an open-ended question “What was the most valuable thing you learned in iCook 4-H?”, youth comments were positive. The theme healthy eating emerged. Youth comments included “Eating cooked apples! They were awesome! The spinach was actually good too,” and “I learned I don’t like bananas, but you should try different foods to be healthy”, showing youth were actively trying, and liking, healthy foods during their iCook 4-H experience. The theme of food safety also emerged with one youth stating, “Always wash your hands before and after so you do not cross contaminate”. Lastly, physical activity was mentioned by youth, with an example: “The most important thing I learned today was exercising my body and how to take my pulse after”. These themes align with the goals of iCook 4-H and encompass the core concepts of cooking (healthy and safely) and playing from the iCook 4-H curriculum.

Almost all adults (93%) reported either strongly agreeing or agreeing that their youth had learned kitchen skills from sessions that would be used at home. Almost 75% stated they were likely or very likely to use the recipes they learned in the sessions. Themes that emerged from open-ended questions regarding “What was the most important part of sessions?”, included quality time together and cooking skills of youth and adult. Quality time quotes were very similar for most adults regarding “Spending time with my child” or “Spending some one on one time with my daughter.” With regards to cooking skills, one said, “Helping my child with knife skills and building her confidence.” And another commented, “Learning to cook because I’m not even close to Martha Stewart level.”

Adults also answered, “What did you think was the most important part of this class for your child?” with similar responses under quality time, cooking skills and healthy eating. Adult comments included: “Getting a mommy and me day and learning how to make a delicious snack”, “Learning about a new vegetable, trying a new recipe that is healthy”, “spending some time with her mom that we don’t often get” and “Quality time together, she wants to keep coming to class every session”. One parent had a child that was both a HSTA teen leader and iCook participant and expressed the value of time with both, “Spending time with my HSTA children and my iCook kid that doesn’t normally occur.” Additionally, the theme of physical activity emerged as well, with one parent stating “[iCook 4-H] helps my child make healthier decisions than I did growing up. The activities were great. Any kind of movement is up and off the couch. They loved the activity with the cups. Just because you are not running laps or walking miles does not mean you are inactive. You are up and moving. Great fun!”. The themes and comments expressed align with all core concepts of iCook 4-H through cooking, eating and playing with an emphasis on togetherness.

### 3.3. HSTA Teen Leader Demographics and Perceptions

Demographic characteristics of the HSTA teen leaders are reported in Table 3. Data on HSTA teen leader demographics were collected by the HSTA program staff prior to the start of the iCook 4-H program and was shared with researchers. Across the eight HSTA programs, teen leaders (*n* = 43) were predominately female (83.7%). Class standing ranged from freshman to senior with the mean age being 15.1 ± 1.2 years. A majority of the teen leaders were white (60.5%).

HSTA teen leaders found the curriculum to be successful and reported that the provided curriculum and training was adequate to teach the session 98% of the time. Open-ended feedback from HSTA teen leaders’ perceptions of the beneficial aspects of sessions for the dyads provided rich insight. When asked “What was the most beneficial aspect of the class for the dyads?”, the themes quality time, healthy eating, cooking skills, physical activity and food safety emerged again with the additional theme of family meals. HSTA teen leaders stated “I feel it gave the parents a fun way to play with their children without wearing themselves completely out. I also believe it provided a lot of the dyads with an easy, tasty recipe.”, “Both the child and the parent are learning new techniques in the kitchen to make their meals healthier and fun” and “The most beneficial aspect was when the child and parent were cooking together because they learned to bond over making healthy meals”. One HSTA teen leader expanded on this adding that iCook 4-H was a good fit as a HSTA project stating, “I thought the whole experience was great for participants. Getting dyads to sit down for a family meal and have dinner time family conversations seemed new for some kids. Kids were timid to start conversations, but it got better. This is a really fun project for HSTA”.

### 3.4. HSTA Teacher Feedback

When prompted “What impact did the iCook 4-H program have on the HSTA teen leaders?”, teachers provided positive comments and discussed the confidence teens gained through leading the program. Themes that emerged were leadership, confidence and responsibility. Concerning increased confidence, one teacher noted, “The students taught iCook which meant they had to teach adults and children thus giving them confidence not only in their presentation skills, but in their knowledge and their ability to manage groups of people.”, with another teacher adding “[the HSTA teen leaders] did the program themselves, they were responsible for its success. These skills are beneficial for them”. The HSTA teachers acknowledged the leadership and responsibility gained, with one of the teachers stating: “The HSTA students called the youth by their names, encouraged them to eat new foods and to try different things. They also shared personal stories with them. It was a great experience for all involved and really brought out the responsibility and leadership in our teenagers!” Further, HSTA teachers reflected the HSTA teen leaders were even making changes in their personal lives, for example, “I think that the teens learned how to prepare new foods and engaged in more family time with their own families. They tell me of the changes they are making in their own lives because of this program”.

However, while most comments were positive, some constructive feedback was provided regarding the design of the program when HSTA teachers were asked “What could be improved in training or curriculum?” Teachers expressed trouble with some of the curriculum content regarding length and required content. One teacher expressed, “I think the transcripts [in the curriculum guides] are great to help the students lead, but they do contain a lot of extra info which I think makes them feel a bit overwhelmed”. Another reflected on the required training videos that used YouTube, stating, “Our problem has been that our students are blocked from YouTube and can’t watch [training] videos. They have had to watch on their phones and that has not been great.” Lastly, one teacher stated the curriculum could be more flexible for high school students in the future and commented, “The recipes are delicious, activities are great, but I think it is too restrictive for high school students to follow protocol exactly. If we do it again, we will modify slightly”.

## 4. Discussion

Based on these results, it was feasible to adapt an adult-led childhood obesity prevention program into a teen led model and participants had a positive perception of the program. Youth behavior frequencies showed slight increase in positive nutrition, physical activity and family meal behaviors suggesting a teen led model can influence youth participant behavior. In addition, qualitative results were positive for the teen led program. Adults reported that their children had learned new skills from the teen leaders during the iCook 4-H sessions. Feedback provided showed that participants found the program to be beneficial to their families through increased quality time with children, learning new recipes and skills and youth being active. Youth echoed the adults’ perceptions of the program through learning healthy eating habits, food safety and the importance of being active. Teen leaders were successful in getting behaviors to translate into the home as a majority of children reported cooking an iCook recipe at home, eating as a family and being active. HSTA teens found the curriculum to be adequate and perceived the sessions as important for families and creating closer family bonds. The HSTA supervising teachers observed the changes in the HSTA teen leaders, reporting their confidence and leadership skills excelled. Further, teachers suggest that leading a COP program impacted the knowledge and decisions the teens were making in their personal lives.

Using teens as leaders has been promoted in recent years and offers a successful alternative to a traditional delivery system of many intervention programs. Using this model allows for peer relationships to be formed which allows for healthy behaviors to be modeled to others, often utilizing the social learning theory framework, in which youth mimic behaviors of others [22]. Through these types of intervention programs, there is more longevity possible as peer or cross-age education flourishes from young people learning and growing from one another as part of everyday life [21]. Further, Ponzio et al. found that teens are effective teachers of youth for numerous reasons including teens relate to and understand children, teens communicate well with children and in return children value teens as teachers [27].

It is unclear why more COP programs are not capitalizing on lifestyle interventions utilizing teens as teachers/leaders. One possible explanation is the perception of the skill level of teenagers. Many adults view teens at a lower level of skill and expertise compared to adults and would be skeptical to promote this avenue of intervention [18], or with many teens having a lack the confidence in their own skillset [44]. Another issue that may prevent programs from utilizing teens as leaders is the intensive training that is required to address the potential lack of skill set, making the demand for a committed, highly skilled adult support essential [44,45].

In addition, there are “essential elements” for successful programming using teen leaders [46,47]. Teens may have limited access to leadership training prior to implementation of a peer led program, therefore, to run a well-organized, successful program, proper training is key. This includes proper teen selection, well-developed curriculum and training materials and appropriate accountability of the teen peer leaders [46]. Strong, consistent, adult support and supervision is also recommended to provide guidance as needed to the teens [46]. For this study, since teens were trained and monitored by HSTA teachers prior to the start of the program, and during the sessions, they were provided with the guidance they needed to be successful.

While this study showed promising results, there were limitations. This study took place exclusively in one state, which leads to lack of generalizability to other locations. Further, HSTA teens were provided training as student researchers and were highly motivated to be involved in this study. Therefore, high school students not trained as HSTA teen leaders might not be as effective. This study was also limited by its small sample of dyad participants, with a large sample size potentially able to provide more insight on the program and teen leader model. Additionally, the self-reported nature of the surveys may have resulted in potential errors as participants could have unintentionally misreported some data. More specifically, self-reported physical activity measures are less dependable than objective measures of physical activity such as with accelerometers [48]. Lastly, as participants were aware they were completing open-ended questions to assess the success of the HSTA teen leader model of iCook 4-H, they might have been biased to respond positively as most adults knew the HSTA teen leaders personally from the local community. The participant responses are subjective and could vary depending on numerous factors.

Future research is needed to expand the study to a larger population sample, in diverse geographical areas. The small sample size may not accurately represent the teenage population. A larger sample size would allow for a broader range of teenage participants representing all types of teen leaders. In the future, researchers might also want to consider collecting more data on the teen leaders, such as anthropometrics as well as biomarkers. This would allow researchers to explore the impact that implementation of a lifestyles approach to obesity prevention has on teen leaders. Further, curriculum materials should be adapted to address the issues expressed by HSTA teachers and teens with extra training and online methods of training as a solution.

## 5. Conclusions

The iCook 4-H program had positive perceptions from the HSTA teachers, HSTA teen leaders and impact on dyads. Based on this study, it is feasible to disseminate iCook 4-H COP programming through well-trained and supervised teen leaders, although further testing is needed in other geographical areas. This approach could serve as a model to implement COP programming while also promoting teen skills and leadership development.

## Figures and Tables

**Table 1:** iCook 4-H curriculum overview.

Session	Session Components		
	Culinary Skills	Physical Activity	Family Meal Time
1	Healthy Snack: Fruit and Yogurt Parfaits	Introduction to iCook 4-H	Family meal journals and technology training
2	MyPlate: Fruit Salsa with Cinnamon Chips	Circle game, intro activity	Components of successful family meals
3	Dairy: Fruit and Vegetable Smoothies	Know your heart rate, using heart and lungs when active	Child parent mealtime dynamics
4	Vegetables: Oven Rasted Vegetables	Charades, resistance training	Place settings
5	Fruits: Baked Apples and Fast Fruit Salad	Stretching and flexibility	Quality communication
6	Grains: Rice Stir-Fry	iCook Shuffle, healthy downtime, sitting less and moving more	Increasing family meal frequency and meal planning
7	Protein: Lentil and Cheese Quesadilla	Cup stacking relay race, group/family games	Avoiding power plays at dinner
8	Creating meals with MyPlate: MyPlate Roll-Up	Traffic light health quiz	Reflection and discussions

**Table 2:** Paired *t*-test of youth behaviors from baseline to post intervention.

Variable—How Often	Baseline Mean (SD) (*n* = 24)	Post Mean (SD) (*n* = 15)	*p*-Value
can use a knife to cut foods	2.17 (0.15)	2.60 (0.19)	=0.0849
can use oven for cooking	1.70 (0.17)	2.07 (0.22)	=0.3334
can cook foods to the right temperature	2.04 (0.15)	2.27 (0.19)	=0.3572
can you use a blender	1.88 (0.13)	2.33 (0.17)	=0.0407 *
can you store foods the right way	2.54 (0.16)	2.47 (0.21)	=0.7774
can you measure ingredients for a recipe	2.63 (0.16)	2.47 (0.20)	=0.5305
can you use MyPlate to plan a meal with all food groups	2.13 (0.17)	2.33 (0.22)	=0.4546
are you physically active for at least 60 min	2.50 (0.14)	2.53 (0.18)	=0.8845
do you have stressful family meals	2.61 (0.14)	2.40 (0.18)	=0.3673
do you help with grocery shopping	1.47 (0.16)	1.53 (0.20)	=0.8311
does your family eat together	2.64 (0.14)	2.60 (0.17)	=0.8678
do you help cook	2.22 (0.15)	2.20 (0.18)	=0.9418
does your family eat without distractions	1.65 (0.17)	2.13 (0.21)	=0.0777
does your family play together	2.09 (0.16)	1.93 (0.20)	=0.5460
do you set healthy goals	1.96 (0.17)	1.87 (0.21)	=0.7456

Paired *t*-test reported in mean and standard deviation of youth behaviors from baseline to post intervention. Scores range from 1–3 with 1 representing never/rarely and 3 representing always/often. * *p* < 0.05.

**Table 3:** Demographic Characteristics of HSTA Teen Leaders.

Characteristic	*n*	%
Grade		
Freshman	24	55.8%
Sophomore	8	18.6%
Junior	7	16.3%
Senior	4	9.3%
Gender		
Male	7	16.3%
Female	43	83.7%
Ethnicity		
White	26	60.5%
African American	8	18.6%
Hispanic	1	2.3%
Biracial	8	18.6%

Grade, gender and ethnicity represented in frequency and percentages.
